# Compendium of *Agrobacterium*-Mediated Tissue Culture Transformation Methods of Various Solanaceae Species

**DOI:** 10.3390/mps8050107

**Published:** 2025-09-11

**Authors:** Caterina Brancato, Najmeh Heusch, Kenneth Wayne Berendzen

**Affiliations:** Plant Transformation Department, Center for Plant Molecular Biology (ZMBP), University of Tübingen, Auf der Morgenstelle 32, D-72072 Tübingen, Germany; caterina.brancato@zmbp.uni-tuebingen.de (C.B.); najmeh.heusch@uni-tuebingen.de (N.H.)

**Keywords:** tissue culture, Solanaceae, tobacco, tomato, potato, *Agrobacterium* transformation

## Abstract

The routine transformation and genetic modification of many plant species other than *Arabidopsis thaliana* is still an arduous task. In many cases, it is necessary to use special tissues or conditions performed under sterile tissue culture conditions. Nevertheless, this approach is often the most expedient one, and streamlining protocols to maximize efficiency and minimize effort without sacrificing quality is paramount to today’s research agendas. The Solanaceae family tends to be amicable to tissue culture and relatively easy to transform. Here, we present our optimized, routine tissue culture protocols for the transformation of *Nicotiana benthamiana*, *Nicotiana tabacum*, *Solanum tuberosum* (potato), and *Solanum lycopersicum* (tomato). We highlight their commonalities and their differences, thus giving the researcher a framework for optimizing their own protocols in their laboratory if needed. Tissue culture transformation is still an important and dynamic field for the advancement of plant research in molecular genetics, physiology, and plant pathology, and will continue to be a viable and important resource into the future.

## 1. Introduction

Plant tissue culture first started in the early 1900s [1]. It has been, and still is, an important component of modern research and agricultural applications since the 1960s. While conventional agriculture is limited to traditional breeding programs, plant transformation and genome editing provide new opportunities to support the clonal propagation and the bio-product industry, basic studies, and crop improvement, and address our current environmental and societal needs. As such, there is continued use and development of transformation protocols of many species, including those well established.

In general, most transformation protocols follow a three-step regeneration process after *Agrobacterium* infection: (1) organogenesis/somatic embryogenesis, (2) shoot induction, (3) root induction [2,3]. The choice of explant tissue affects the transformation efficacy. For example, most protocols typically experiment with leaves, stems, hypocotyls, and cotyledons, but other tissue types are explored as well (see various references herein and our protocols below). A meristem is thought to originate from a single cell and represent a clonal process [4,5]. When the hormones auxin and cytokinin are present at the correct balance for each species and cultivar, it is possible to spontaneously generate shoots directly through a process called somatic embryogenesis, as the tissue that does this goes through an embryonic-like growth phase [4,6]. Organogenesis, on the other hand, results in either callus (an undifferentiated tissue state), shoots only (often due to root developmental suppression by high cytokinin, or roots only (often occurring spontaneously or in the presence of low auxin in the absence of exogenous cytokinin) [4].

The transformation method of choice used here is *Agrobacterium* infection. Agrobacteria have evolved to inject single-stranded DNA into plant cells, the transferred DNA or T-DNA, which integrates into the plant genome. This integration is facilitated by bacterial proteins that also interact with native plant proteins, and natively, the T-DNA produces auxins and cytokinins that cause the tissue to become oncogenic and produce callus that exudes amino acids (opines), which the bacteria consume: the crown gall disease [7,8]. Studying *Agrobacterium* infection led to the discovery that these oncogenes could be removed and any DNA within the transfer region could be transferred by *Agrobacterium* into plant cells, simplifying the job of genetic transformation. This is now a standard laboratory practice in recombinant technology. Importantly, the natural infection sites are those where plant tissue is wounded, and the wounded surfaces are those that are transformed in all transformation protocols as well [2,3,9,10].

*Nicotiana tabacum* and *Nicotiana benthamiana* have been model species for more than 40 years [11]. *N. tabacum* was one of the first species to be utilized for tissue culture ([12] and references herein) and has many applications in the biopharmaceutical and biofuel industries [13]. *N. benthamiana* is an Australian relative of *N. tabacum*. *N. benthamiana* is susceptible to over 500 viruses, including some restricted to monocots, and even Flock house animal virus, which incidentally can also infect some Brassica and monocot crop species [14]. This sensitivity in *N. benthamiana* has been tied to a disruption of an Rdr1 DNA-dependent RNA polymerase [15]. Both species have found a niche in modern-day research. *N. benthamiana* is also very popular for the bio-production of proteins by transient Agro-transformation of leaf cells [16], a technique used to study basic science questions as well [17].

Tomato (*Solanum lycopersicum*) is an important food crop around the world. A native of South America, it was eventually brought to Europe in the 1500s and North America in the 1800s [18]. It is a perennial plant that has both determinate and indeterminate growth. Tomato can be propagated by seed or clonally by tip or shoot cuttings [19,20] and has many readily available resources like mutant populations, high-quality reference genomes, and bioinformatic tools [21,22].

Potato is a major caloric worldwide food source, with hundreds of tons being produced annually. Breeding is complicated as it has a highly heterozygous genome that hinders seed-based propagation programs ([23] references therein). The cultivar Désirée is a red-skinned tuber, a late-season variety, originally bred in the Netherlands in 1962 by crossing cv. Urgenta with Depesche; it has favorable economic traits and high tolerance to drought and some pathogens ([24] references therein). The haplotype genome assembly of Désirée has been solved and is available [24]. Interestingly, the US potato Genbank cultivar from 2015 turned out to be a somatic mutant of cv. Urgenta [25]; thus, researchers are advised to check their germplasm source.

In this compendium, we bring together three protocols for the transformation of various Solanaceae genera and species that we have used over the last 25 years. This work is in various master’s and doctoral theses, and a cornerstone of various publications as acknowledgements or authorship [26,27,28]. The goal has been, and is, the continual optimization and simplification of the steps needed for smooth and reliable tissue culture transformation as part of our Institute’s research objectives. These protocols are rooted in works that showed that many genera and species from Solanaceae could be transformed [5,29,30,31,32], and observations we have made over the years that work best with our equipment and research paradigm. Most importantly, we wish to provide the readers with a good base to start their own tissue culture program and provide a rich resource for those who already have one.

## 2. Experimental Design

**Figure 1:**
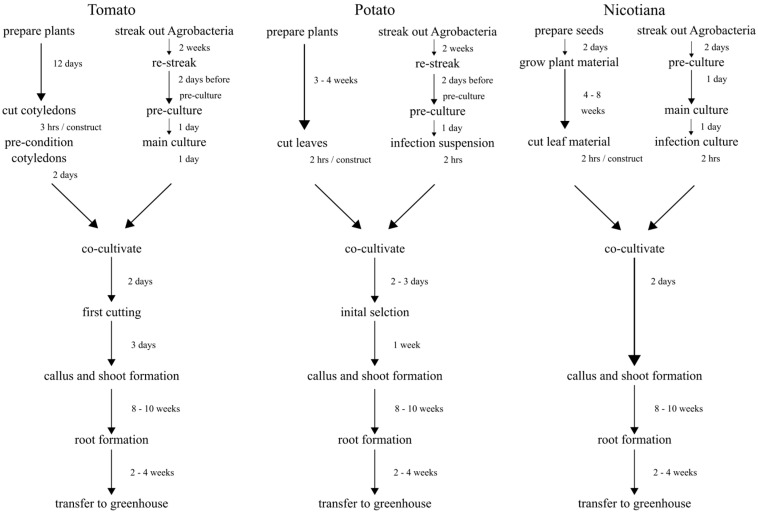
Schematic workflow overview for all three protocols.

While there are similarities to all three protocols, they are still tailored to each genus. Here is a general workflow overview (Figure 1) illustrating ideal time frames to obtain the first rooted plant. The duration and efficiency are always dependent on the gene of interest and the genotype. Thus, transformations usually take at least the minimum shown here, but on occasion can last up to 6 months.

### 2.1. Chemicals

- Murashige & Skoog (MS) Salts (Duchefa, Netherlands; Cat. no.: M0221).
- Daishin Agar (Duchefa, Netherlands, Cat. no.: D1004; CAS: 9002–18–0). Note: density ≥ 1000 g/cm^2^.
- Naphtalenacetic acid (Duchefa, Netherlands; Cat. no.: N0903; CAS: 86–87–3).
- Acetosyringone (Merck, Germany; Cat. no.: D134406, CAS: 2478–38–8).
- Amphotericin B fungicide (Merck, Germany; Cat. no.: A2411; CAS: 1397–89–3).
- Cefotaxime sodium anti-bacterial (Duchefa, Netherlands; Cat. no.: C0111, stored at 4 °C).
- Basta herbicide (CAS: 77182–82–2, 183 g/L Glufosinate; liquid, stored at RT). Note: as applicable, check for regional law restrictions.
- 6-Benzylaminopurine (Duchefa, Netherlands; Cat. no.: B0904; CAS: 1214–39–7).
- Indole-3-acetic acid (Merck, Germany; Cat. no.: I2886; CAS: 87–51–4).
- Gibberellic acid 3 (GA_3_) (Duchefa, Netherlands; Cat. no.: G0907; CAS: 77–06–5).
- Hygromycin B (Duchefa, Netherlands; Cat. no.: H0192; CAS: 31282–04–9).
- Kanamycin monosulfate (Duchefa, Netherlands; Cat. no.: K0126, CAS: 25389–94–0).
- Ticarcillindisodium/potassium-clavulanate (Duchefa, Netherlands; Cat. no: T0190).
- trans-Zeatin (Duchefa, Netherlands; Cat. No.: Z0917, CAS: 1637–39–4).
- Vancomycin hydrochloride (Duchefa, Netherlands; Cat. no.: V0155, CAS: 1404–93–9).
- Zeatin riboside (Duchefa, Netherlands; Cat. no.: Z0937, CAS: 6025–53–2).

### 2.2. Equipment

- Standard laboratory equipment:
  Petri dishes, pH meter, hot plate with magnetic stirrer, analytical balance, refrigerator, freezer, water purification system, autoclave, spatulas, forceps, laminar flow hood, and plant cultivation chambers (soil-free).
- Canning Jars type “Sturz-Gläser” ¼ Liter (height: 7 cm; top-diameter: 10.5 cm) and ½ (height: 11 cm; top-diameter: 10.5 cm) Liter (Weck, Bonn, Germany): Baked-sterilized: 180 °C for 10 h (Binder Heating Chamber).
- Razor-blade holder and beard-shaving quality razor blades (a very sharp edge is essential).
- Tea ball sieves wrapped in aluminum foil and autoclaved.

## 3. Procedures

### 3.1. Cleanliness and Working Aseptically

One of the most challenging aspects of working with tissue culture is that it is absolutely essential to maintain a continued, clean, and infection-free working environment throughout the entire span of each experiment. Tissue culture transformation of the various Solanaceae species given here takes on average 2 to 4 months before the plants can be delivered to the greenhouse. Therefore, we strongly recommend that you routinely keep your laminar flow hoods clean, change their filters regularly, and immediately sterilize them after spills or accidents. Similarly, your plant cultivation chambers should be maintained under strict usage restrictions: no soil; do not allow people to travel from greenhouses to climate chambers; do not open contaminated material and remove it immediately; and routinely clean all surfaces, floors, and exchange air filters regularly. Furthermore, the presence of antibiotics does not prevent the growth of fungal contaminants, which are much tougher, if not sometimes impossible to eliminate. Therefore, we suggest you learn and implement classical microbial sterilization techniques: work with flame to sterilize metalware directly before use, heat up vessel spouts before removing or dispensing liquids to other dishes, be aware of how your laminar flow hood works, and know where to place your hands and forearms as not lead to contamination. Practicing these techniques will lead you to enjoying and maintaining a successful tissue culture laboratory.

### 3.2. Sterilization of Glass- and Metalware

Glassware/culture vessels should ensure aseptic techniques, being able to prevent direct access to ambient air. After testing, we found the Weck glassware can be washed in a laboratory dishwasher and sterilized by just baking at 180 °C for 10 h. This provides us with an economical, sustainable resource. If you are new to tissue culture, you should test which material and procedure works best for you to maintain sterility. Metalware should be clean, wrapped in at least one and up to three layers of aluminum foil, which is folded in a way that seals and is yet easily undone under the clean bench safely and easily. Once unwrapped, you may use 70% ethanol to clean them, but all cutting (razor blades) and transfer surfaces (tweezers) should still be flame sterilized directly before use. Make sure you let the metal cool down to room temperature before handling any plant material.

### 3.3. Working with Agrobacteria

Various strains of Agrobacteria can be used for transformation. In general, we have overwhelmingly used nopaline strain GV3101 (cured C58 background C58C1) [33] harboring helper plasmids pMP90 or pMP90RK, which have the T-region from Tumor Inducing (Ti) plasmid pTiC58 substituted with gentamycin, or with mini-RK2 replication machinery added alongside kanamycin resistance, respectively [34]. We have used GV3101 with various binary vectors over the years (e.g., pBIN, pCAMBIA/pPZP, pPGTV, and derivatives) [7,8,35,36]. It is essential that the researchers ensure that their bacteria are carrying both the binary vector and essential helper plasmid(s) (e.g., pMP90RK for RK2-mini origins [34] or pSOUP for pGREEN binary plasmids [35], otherwise, T-DNA transfer cannot occur. We have also had success with GV2260 (C58 background with the T-region from Tumor Inducing (Ti) plasmid pTiB6S3 deleted and substituted by pBR322 [37].

### 3.4. Climate Chamber Conditions

We found that we can use the same climate chamber conditions for all four species to good effect. This saves us in both space and coordination. Therefore, we have discovered a light cycle of 13 h light/11 h dark with alternating fluorescent lamps Osram L 18 W/840 LUMILUX and Osram L 18 W/77 FLUORA side-by-side to provide the best spectral mix for promoting plant growth. The radiant flux is between 50 and 60 µmol s^−1^ m^2^ when our jars are about 50 cm from the light sources. Temperature: constant 23 °C; humidity: 60%.

### 3.5. Stable Tissue Culture Transformation of Solanum Lycopersicum (Tomato) ‘Moneymaker’

#### 3.5.1. Seed Sterilization (1 h)

**Figure 2:**
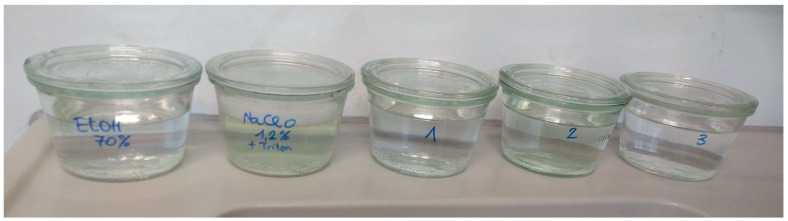
Vessels for seed sterilization and passaging as described above.

**Figure A1:**
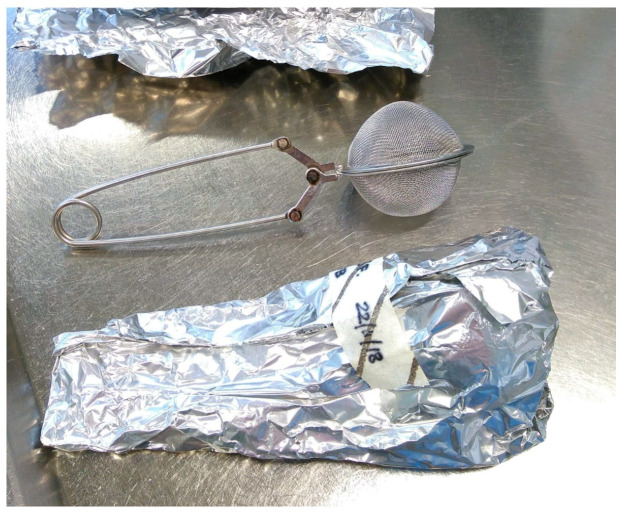
Stainless steel mesh tea ball sieves wrapped in aluminum foil and autoclaved.

Two weeks before Agrobacteria co-cultivation (Section 3.5.4), surface-sterilize about 300 seeds, approx. 0.5 g *Lycopersicon esculentum* ‘Moneymaker’, in autoclaved stainless steel mesh tea ball sieves (Figure A1). Seeds can be purchased from a seed supplier or self-harvested from a greenhouse. Working under the laminar flow hood, passage the seeds in the tea ball sieves in sterile jars (Figure 2) sequentially by the following process:

- Swirling for 3 min by hand in 200 mL 70% ethanol.
- Swirling for 10 min by hand in 200 mL 1.25% sodium-hypochlorite containing a few drops of 0.001% Triton X-100 (in sterile water).
- Rinsing by passaging in 3 × 200 mL for 30–60 s each in sterile double-distilled or ion-exchanged deionized water.
- Remove the seeds from the tea sieves by depositing them into a dry, sterile jar.
- Let the seeds dry under the laminar flow hood overnight.
- Store the dried, sterile seeds in the same jar for two days in the dark at 4 °C.

#### 3.5.2. Preparing Cotyledon Material for Transformation

1. Climate chamber conditions: see Section 3.4.
2. Sowing the seeds (1 to 3 h)

A total of 12 days before co-cultivation (Section 3.5.4), sow up to 30 seeds per jar on the Tomato Medium (ToMe, Section 5.3.2). In total, 10 jars are enough to obtain about 400 suitable cotyledons.

Germinate the seeds in the dark at 22 °C.

**Figure 3:**
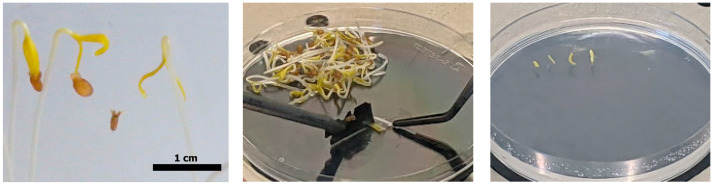
Cutting the cotyledons (12 days old): (**left**) close-up of the cotyledons, (**center**) adding the incision, and (**right**) after transfer to ToCM.

**Figure A2:**
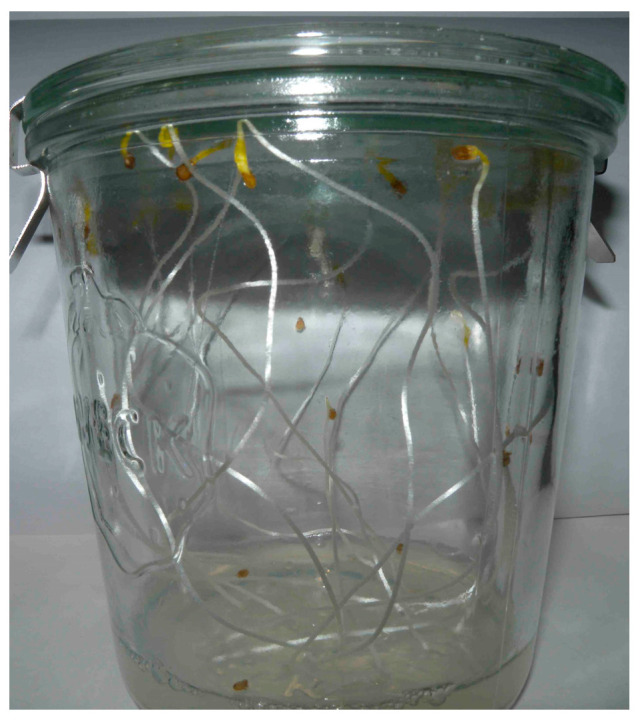
Twelve-day-old etiolated tomato seedlings.

We recommend beginning on a Friday to plan for weekends/off days.


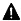
 **CRITICAL STEP** The seeds should germinate in absolute darkness. The cotyledons should be about 1 cm long, still etiolated, and the first true leaves not yet developed (see also [19,20]). See Figure 3 and Figure A2.

3. Isolating the cotyledons for transformation (3 h/construct plus 2 days)

Plan 3 h of work per construct to be transformed.

If you started on a Friday, this step should fall on a Wednesday.

- Apply a few drops of the Tomato Liquid Germination Medium (ToLGM, Section 5.3.3) onto a sterile Petri dish so that the plant material does not dry out while cutting it.
- Excise the cotyledons from the seedlings by cutting directly below the leaves and discarding the rest.
- Remove the top and base of the leaf and make a small incision along the long axis near the center of each leaf (Figure 3).

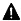
 **CRITICAL** Do not squeeze or crush the cotyledons.

- Transfer the prepped cotyledons onto the Tomato Conditioning Medium (ToCM, Section 5.3.4) with the adaxial (upper) surface down, in full contact with the medium. Usually 10 Petri dishes are enough, with each dish holding about 20 cotyledons with enough space between them and the walls (Figure 3). If a Petri dish does become contaminated, discard it immediately.
  **OPTIONAL**: If possible, use only cotyledons that are not rolled, as they are very difficult to lay out on the selection plates and need to have good contact with the media to be selected properly.

- Incubate the cotyledons for 2 days in absolute darkness at 22 °C.

#### 3.5.3. Preparing the *Agrobacterium* Suspension for Tomato

1. Plate growth of Agrobacteria (2 h)
  Streak out the Agrobacteria on YEB-plates (Section 5.1.1) with the appropriate selection antibiotics roughly at the same time you start sowing the seeds (Section 3.5.2 step 2). Re-streak them two days before cutting the cotyledons (Section 3.5.2 step 3). This should fall on the Monday after the Friday you started cutting.

2. Pre-culture liquid growth of Agrobacteria (1 day)
  Inoculate 3 mL low-salt LB (ls-LB, Section 5.1.2) with one single colony with the appropriate selection antibiotics. Shake overnight at 180 rpm at 28 °C. This should fall on the Wednesday you started cutting the cotyledons on (Section 3.5.2 step 3).

3. Agrobacteria infection culture (1 day)
  One day before co-cultivation (Section 3.5.4), inoculate 1 mL of the pre-culture (Section 3.5.3 step 2) into 100 mL bacterial growth medium (BGM; Section 5.1.3) with the appropriate antibiotics. Shake overnight at 180 rpm at 28 °C. This should fall on the Thursday after you cut the cotyledons on (Section 3.5.2 step 3).

#### 3.5.4. Transformation: Infection and Co-Cultivation of Plant Material with Agrobacteria

This step should occur on the Friday after you have cut the cotyledons (Section 3.5.2 step 3).

1. Bring the Agrobacteria to the infection concentration (2 h)

- Centrifuge the infection culture (Section 3.5.3 step 3) for 10 min at 3000× *g*, RT.
- Resuspend the bacteria in 10 mM MgSO_4_ with 200 µM acetosyringone and adjust the OD_600_ to 1.0, in a final volume of 50 mL.

2. Co-cultivate Agrobacteria with the wounded cotyledon tissue (2 days)

- Dispense about 1 drop to each cotyledon with a 1000 µL pipette. Total volume should roughly be 2 mL per Petri dish.
- Incubate the co-cultivation for 2 days in the dark at 22 °C.

#### 3.5.5. Selection of Transformed Tissue

1. Climate chamber conditions: see Section 3.4
2. Initial transfer to selection medium (3 days)

- Transfer the cotyledons adaxial (upper) side up to small canning jars (1/4 L) with the Tomato Selection Medium (ToSM, Section 5.3.5). This should fall on the Monday after co-cultivation (Section 3.5.4 step 2). 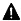 **CRITICAL** If you are selecting with Kan, use 35 mg/L at this point. For other antibiotics, see the Reagent Setup Section.
- Incubate for 3 days in the climate chamber.

3. Sub-culturing for shoot and callus isolation (8 to 10 weeks)

- Transfer the cotyledons to fresh ToSM in small canning jars (1/4 L) once a week. 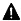 **CRITICAL** If you are selecting with Kan, use 50 mg/L at this point for two weeks, and thereafter 100 mg/L Kan.
- After a couple of weeks, the first callus and some with shoots should form.
- Once identifiable, place callus and shoots in direct contact with ToSM.

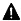
 **CRITICAL** Shoots are to be cut near the base; avoid any old or callus-like tissues.

**RECOMMENDATION**: Naming scheme. We recommend that you assign each callus an individual number and that the shoots that derive from those calli get child assignments, i.e., Callus 1, Shoot 1 (e.g., 1.1, 1.2, 1.3, etc.), as we anticipate that each callus is likely from a single T-DNA integration event. This will help you in collecting independent insertion events to control for biological variance.

#### 3.5.6. Regeneration of Transgenic Tomato Plants (2 to 4 Weeks)

After approximately two months, shoots can be cut and transferred to the Tomato Rooting Medium (ToRM, Section 5.3.6) in large canning jars (1/2 L). We recommend that you place around 4 plants per jar. 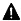 **CRITICAL** If you are selecting with Kan, use 20 mg/L. **OPTIONAL**: If you have any trouble with rooting, try leaving out the selection antibiotic.

**Figure A3:**
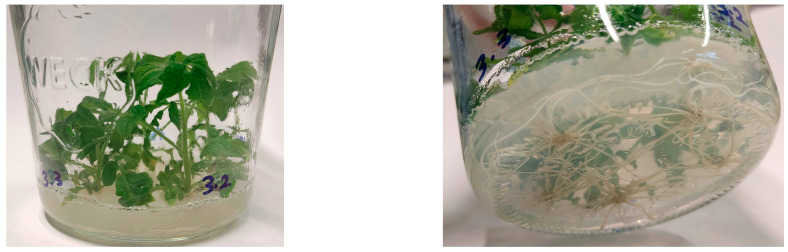
Tomato plants ready to be moved to the greenhouse.

- The callus should be kept, as more shoots will form over time. You can discard calli that lose their vitality (yellow or brown). This is useful in case it is difficult to readily identify transgenic plants.
- Do not let the shoots touch the top of the jars to maintain axenic culture. You may remove the top of the plants and transfer the cuttings to fresh ToRM.
- You can bring the plants to the greenhouse as soon as the roots are well visible and moderately extensive (~1 to 3 cm long with lateral roots, see Figure A3).
- Tomato plants take up a lot of space and take time to flower and set seed; therefore, we advise that you test the transgenic status of each plant as soon as possible in order to preserve greenhouse space and assay if the transformation vector was performing as desired.

### 3.6. Stable Tissue Culture Transformation of Solanum Tuberosum (Potato)

#### 3.6.1. Plant Material (Continuous, Every Four Weeks)

- You will need about 25 sterile plants, three to four weeks old

**Figure 4:**
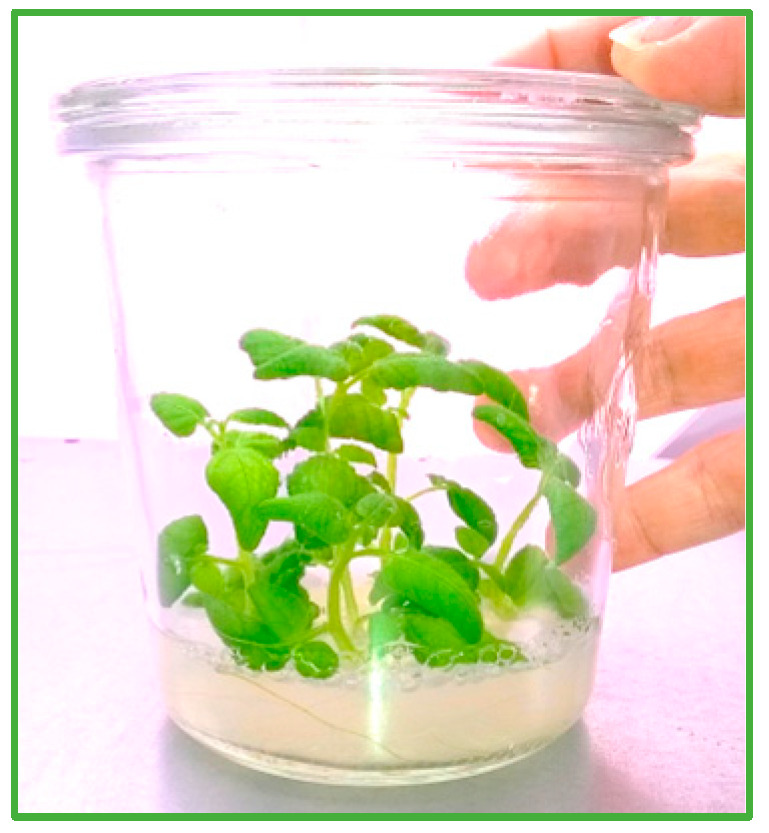
In vitro cultivated potato at three weeks.

For our protocol, we maintain sterile shoots of potato *Solanum tuberosum* cultivar Désireé in tissue culture (Figure 4) by propagating not more than 5 plants (shoot apex with 2 to 3 fully expanded leaves; [19]) every four weeks to new jars on solid MS Medium with 2% sucrose (PoSMS, Section 5.4.2). It is important to prevent the tops of the shoots from touching the lids.

We use the same light conditions for propagation and selection, as described in Section 3.4.

**OPTIONAL**: If you do not have access to a potato maintained in tissue culture, you can request some from a known laboratory or make your own from auxiliary buds formed on tubers [31,38]. You will want to guarantee that they are virus-free [31,39]; i.e., we have a certificate from a local Agriculture and Food Safety Protection Office that stipulates our potato line is Potato Spindle Tuber Viroid free.

#### 3.6.2. Preparing the *Agrobacterium* Suspension for Potato

1. Plate growth of Agrobacteria (2 days)

Streak out the Agrobacteria on YEB-plates (Section 5.1.1) with the appropriate selection antibiotics (Section 3.3) on a Saturday and incubate them at 28 °C for two days.

2. Pre-culture liquid growth of Agrobacteria (1 day)

Inoculate 5 mL YEB medium (Section 5.1.1) with one single colony with the appropriate selection antibiotics. Shake overnight at 180 rpm at 28 °C. This should fall on the Monday after you struck out the bacteria (Section 3.6.2 step 1).

3. Agrobacteria infection culture (2 h)

On the Tuesday after Section 3.6.2 step 2, collect the bacteria from the pre-culture by centrifugation at 3000× *g* for 3 to 5 min and resuspend the pellet in 30 to 100 mL YEB medium without antibiotics. Check that the OD_600_ is between 0.6 and 1.0.

#### 3.6.3. Transformation: Infection and Co-Cultivation of Plant Material with Agrobacteria (2 h/Construct Plus 2 to 3 Days)

- Dispense 10 mL of liquid MS Medium (PoMS, Section 5.4.2) into 8 Petri dishes (9 cm Ø).
- Excise leaves from the potato tissue culture, immediately remove the petiole and a bit of the leaf base, and apply two tiny transverse cuts to the mid-vein of the abaxial surface.
- Plan for two hours per construct for leaf excision.
- Place the cut leaf in the liquid PoGM (Section 5.4.3), adaxial side facing down, directly in contact with the medium.

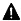
 **CRITICAL** The leaves are very sensitive to any kind of injury, i.e., being burned or handled too harshly. Therefore, never squeeze the tissue too hard, and let the forceps cool down to room temperature after flame sterilization. Use very sharp razor blades to avoid microscopic bludgeoning damage. Finally, use healthy, vibrant, green leaves from the top of the plants.

**Figure A4:**
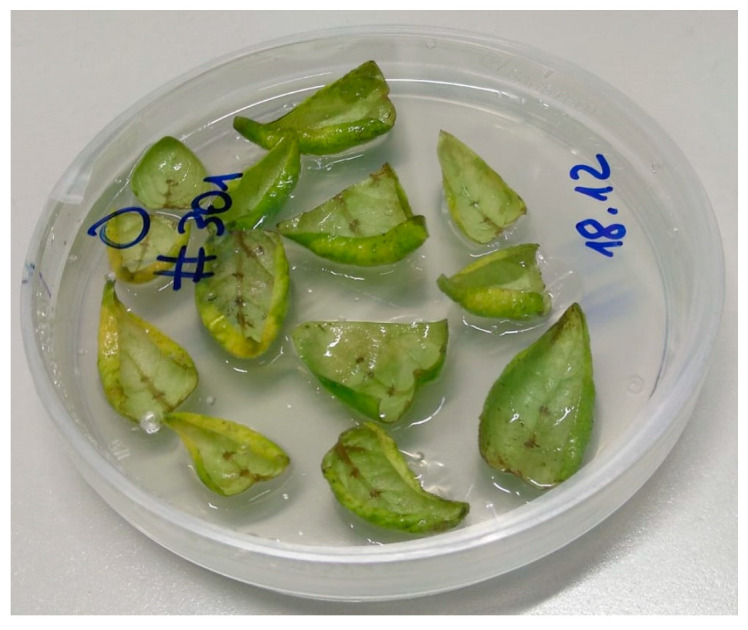
Potato leaves prepared for infection.

- You should prepare around 100 leaves in total, roughly 12 to 15 leaves per Petri dish (Figure A4).
- Apply 50 µL of the *Agrobacterium* infection culture (Section 3.6.2 step 3) directly to the Petri dish and shake lightly for 3 to 5 min on a shaker.
- Incubate the co-culture plates in the dark at 22 °C for 2 to 3 days.

#### 3.6.4. Selection of Transformed Tissue (8 to 10 Weeks)

**Figure A5:**
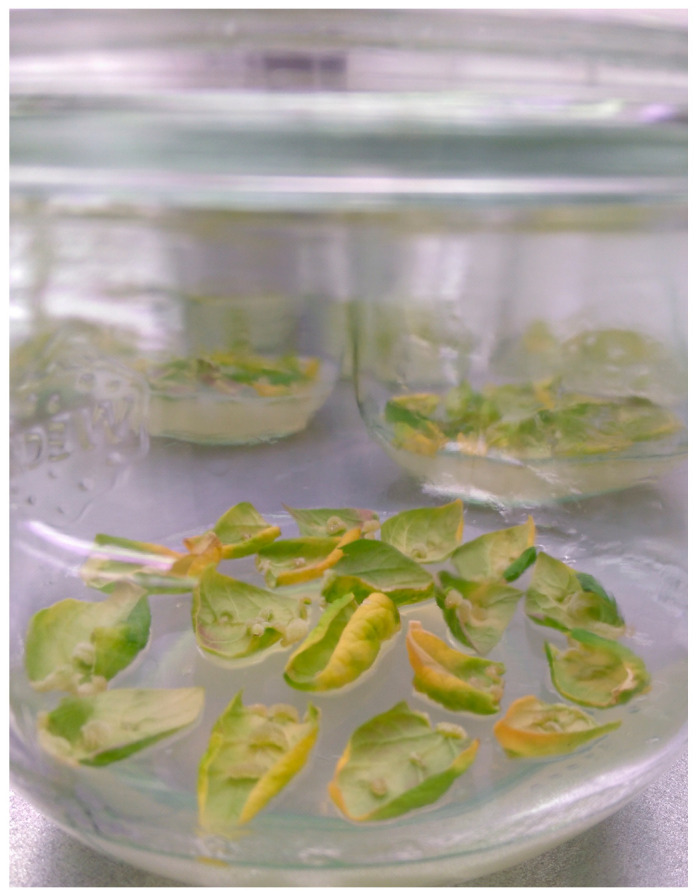
Potato leaves after co-cultivation and placed on poCIM.

1. Transfer the leaves to the Potato Callus Induction Medium (PoCIM, Section 5.4.4), adaxial side facing down, in direct contact with the medium (Figure A5).

- After one week, transfer the leaves to the Potato Selection Medium (PoSM, Section 5.4.5), adaxial side facing down, in direct contact with the medium.

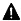
 **WARNING** Leaves and the jars with contaminated with fungi are best just discarded. **OPTIONAL**: Uninfected leaves adjacent to infected material can be transferred to PoSM with fungicide amphotericin (Section 5.2.1) and sealed with Parafilm.


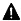
 **WARNING:** Leaves with a strong *Agrobacterium* infection should also be discarded. **OPTIONAL**: As above, try removing uninfected material to fresh jars, and you can try washing infected leaves with 10 mM MgSO_4_ plus 250 mg/L cefotaxime (Section 5.2.2) and isolate them from healthy tissue in their own, separate jars. If necessary, this can be performed every few weeks; washing is more effective than just adding it to the medium.

- Transfer to fresh medium every 10 days until callus appears or all the tissue dies. **OPTIONAL**: As long as there are no shoots, you may perform this and all of the above steps in Petri dishes if desired.

2. Callus isolation and cultivation

- As soon as you can clearly identify callus forming, you should move the callus to separate jars for further cultivation by cutting them free from any attached leaf tissue.
- Continue to transfer the callus every 10 days and expect shoot formation in a few weeks.

#### 3.6.5. Regeneration of Transgenic Potato Plants (2 to 4 Weeks)

**Figure A6:**
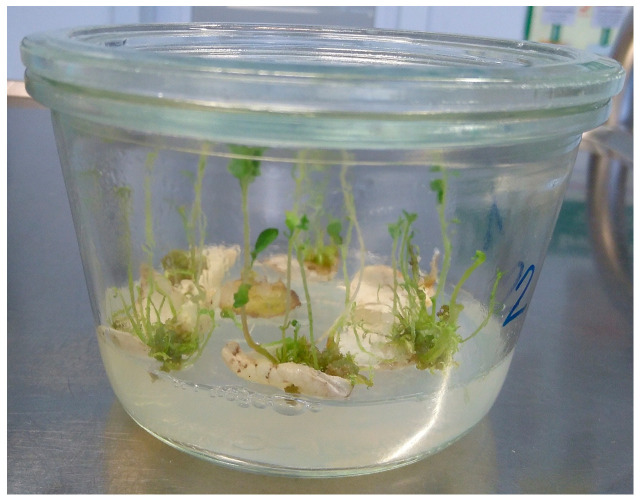
Potato shoots forming from callus.

- After a couple of weeks, the first shoots should form. The shoots will be delicate and thin, but vital (Figure A6). Fresh shoots should not be allowed to touch the top of the jars to maintain axenic culture. They should be excised and moved to new jars. As they get taller, move them to larger jars (1/2 L).

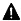
 **CRITICAL** Shoots are to be cut near the base; avoid any old or callus-like tissues.

- After approximately 2 months, shoots that are green and have at least one to two leaves can be cut and transferred to the Potato Rooting Medium (PoRM, Section 5.4.6). Keep in mind that potato shoots coming out of the calli will be thin and delicate (Figure A6).
- You can bring the plants to the greenhouse as soon as the roots are visible and minimally extensive. As with the shoots, the roots will be thin and fine.
- The callus should be kept, as more shoots will form over time. You can discard calli that lose their vitality (yellow or brown). This is useful in case it is difficult to readily identify transgenic plants.

**RECOMMENDATION**: Naming scheme. We recommend that you assign each callus an individual number and that the shoots that derive from those calli get child assignments, i.e., Callus 1, Shoot 1 (e.g., 1.1, 1.2, 1.3, etc.), as we anticipate that each callus is likely from a single T-DNA integration event. This will help you in collecting independent insertion events to control for biological variance.

### 3.7. Stable Tissue Culture Transformation of Nicotiana Benthamiana and Nicotiana Tabacum

This protocol works equally well for both Nicotiana benthamiana and Nicotiana tabacum ‘Samsun NN’ in our hands. Please pay attention to when there are specific differences between the two when mentioned in the protocol.

#### 3.7.1. Seed Preparation (2 Days)

**Figure 5:**
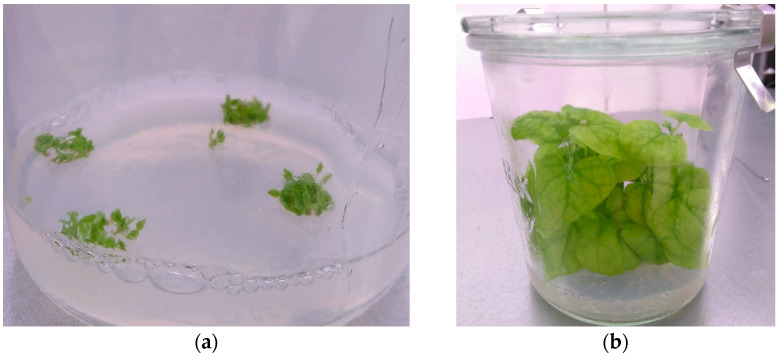
Growth of *Nicotiana benthamiana*: (**a**) *N. benthamiana* 1 week old (**b**) and 7 weeks old.

1. Surface-sterilize 10 to 25 seeds by placing them in Eppendorf tubes. Working under the laminar flow hood, passage the seeds by the following process:
  - Add 1 mL 70% ethanol and place on a shaker for 3 min. Decant or remove by pipetting.
  - Add 1 mL of 1.5% sodium hypochlorite containing a few drops of 0.001% Triton X-100 and place on a shaker for 8–10 min. Decant or remove by pipetting.
  - Wash with 1 mL for 1 min in sterile double-distilled or ion-exchanged deionized water. Decant or remove by pipetting.
    ○ Repeat this wash step two more times.
  - Resuspend the seeds in 0.1% agarose (Section 5.5.1) and distribute on *Nicotiana* Growth Medium (NicoGM, Section 5.5.2) in jars or Petri dishes. You may find with practice that you can dispense the seeds just in pure, sterile water.
  - Place the jars in the dark and cold (4 °C) for 2 days.
2. Bring the vernalized seeds to your cultivation chamber.
3. Grow N. *benthamiana* for 7–8 weeks; you need 6 plants per transformation (example in Figure 5).

Grow *N. tabacum* for 4–6 weeks; you need 2 plants per transformation.

#### 3.7.2. Preparing the *Agrobacterium* Suspension for *Nicotiana*

1. Plate growth of Agrobacteria (2 days)

Streak out the Agrobacteria on solid LB-plates (Section 5.1.2) with the appropriate selection antibiotics (Section 3.3) on a Saturday and incubate them for 2 days at 28 °C.

2. Pre-culture liquid growth of Agrobacteria (1 day)

Inoculate 3 mL liquid low-salt LB (ls-LB, Section 5.1.2) with one single colony with the appropriate selection antibiotics. Shake overnight at 180 rpm at 28 °C. This should fall on the Monday after Section Plate Growth of Agrobacteria (2 days).

3. Agrobacteria infection culture (1 day)

The next day, inoculate 2 mL of the pre-culture (Section 3.7.2 step 2) into 250 mL LB medium with the appropriate antibiotics. Shake overnight at 180 rpm at 28 °C. This should now fall on a Tuesday.

#### 3.7.3. Infection and Co-Cultivation of Plant Leaves with *Agrobacterium* (2 Days)

1. Collect the bacteria from the infection culture (Section 3.7.2 step 3) by centrifugation at 3000× *g* for 5 to 10 min and resuspend the pellet in sterile 10 mM MgCl_2_ without antibiotics; adjust the OD_600_ to 1.0, in a final volume of 100 mL. This should now fall on a Wednesday.
2. Place ~30 mL of the suspension into 2 to 3 sterile Petri dishes (Ø 9 cm). You will be dipping the leaf explants into the *Agrobacterium* solution.
3. Cut the leaves into about 30–35 square pieces per sterile Petri dish, avoiding the central vein:
  - For *N. tabacum* 0.5 × 0.5 cm squares
  - For *N. benthamiana* 1 × 1 cm squares

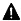
 **CRITICAL** Use green and healthy leaves. Do not let the leaf pieces overlap.


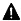
 **CRITICAL** The leaves are sensitive to any kind of injury; if burned, singed, or handled too harshly, the tissue will die. Therefore, never squeeze the tissue and make sure to let the forceps cool down to RT after flame sterilization. Try to use very sharp razor blades to mitigate bludgeoning damage.

4. Incubate the explants abaxial-side down in the bacteria suspension for 3 min with gentle shaking.
5. Transfer the explants to Nicotiana Growth Medium (NicoGM, 5.5.2), abaxial surface down, in contact with the medium.
6. About 100 pieces are enough for one transformation (about 3 Petri dishes).
7. Incubate them for 2 days in the dark at RT.
8. At any time during the procedure, if you see excessive Agrobacteria growth on the explant tissue, exchange the plates with fresh ones before proceeding.

#### 3.7.4. Selection of Transformed Callus Tissue (Typically 3 to 6 Weeks)

1. Transfer leaves to Nicotiana Selection Medium (NicoSM, Section 5.5.3) in small jars. This should fall on a Friday after co-cultivation (Section 3.7.3 step 7).

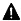
 **CAUTION** At the beginning, leaves with strong *Agrobacterium* infection should be outright discarded or washed in 10 mM MgSO_4_ containing 250 mg/L cefotaxime and incubated separately on NicoSM plates, away from the other explants. If necessary, this can be performed every few weeks; washing is more effective than just adding it to the medium.

2. The leaves must be transferred to fresh NicoSM medium every 7 to 10 days.

- As soon as you can clearly identify callus forming, you should move the callus to separate jars by excising it free from any attached leaf tissue. Maintain the callus on NicoSM every 10 days as well.

**RECOMMENDATION**: Naming scheme. We recommend that you assign each callus an individual number and that the shoots that derive from those calli get child assignments, i.e., Callus 1, Shoot 1 (e.g., 1.1, 1.2, 1.3, etc.), as we anticipate that each callus is likely from a single T-DNA integration event. This will help you in collecting independent insertion events to control for biological variance.

#### 3.7.5. Regeneration of Transgenic Plants (Approximately 2 Months)

**Figure 6:**
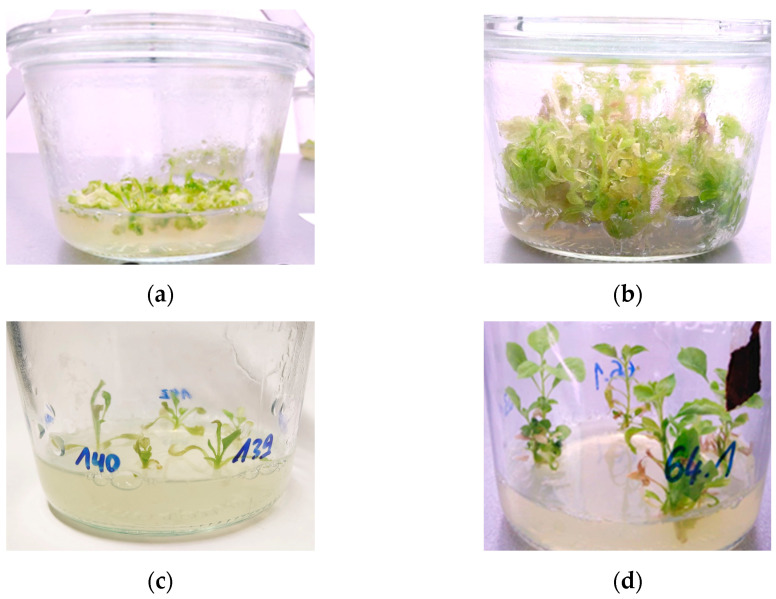
*Nicotiana benthamiana* shoots: (**a**) Shoots initials forming on callus (ca. 3–6 weeks). (**b**) Shoots reading for cutting (ca. 4–8 weeks). (**c**) Size of shoots when placed on NicoRM (ca. 4–8 weeks). (**d**) Shoots ready to be sent to the greenhouse (ca. 2–4 months).

- After a couple of weeks, shoots should begin to form (Figure 6a). Transfer the callus with shoots every 10 days on NicoSM until they are roughly 1 to 2 cm tall (Figure 6b).
- Now the shoots should be large enough to be excised and moved to large jars with *Nicotiana* Rooting Medium (NicoRM, Section 5.5.4; Figure 6c).
- Move the plants to the greenhouse once they have several branches, leaves, and roots that are visible and moderately extensive (Figure 6d).
- The callus should be kept, as more shoots will form over time. You can discard calli that lose their vitality (yellow or brown). This is useful in case it is difficult to readily identify transgenic plants.
- Do not let the shoots touch the top of the jars to maintain axenic culture. You may remove the top (2 to 3 cm) of the plants and transfer the cuttings to fresh NicoRM.

## 4. Expected Results, Notes, and Discussion

For all the protocols, you should be able to pass through all three stages: organogenesis/somatic embryogenesis, shoot formation, and then root formation. In the end, you should be able to obtain plants that have leaves, produce roots, and have survived the selective media. Sometimes there are “escapes”. Escapes are plants that survive the full transformation procedure but are non-transgenic. Therefore, successful genetic transformation events must be confirmed by protein and DNA analysis, for example, immunoblotting or PCR-genotyping, respectively. Keep in mind that transformation sometimes can be dependent on what gene is being expressed and, therefore, also influences the transformation efficiency. Ranges from 15% to 95% are acceptable, with at least 55% being a realistic expectation. It is best to screen as soon as possible in order not to fill up the greenhouse with many plants that take up a lot of space.

Many of the original protocols used “nurse feeder layers”, which were cell suspension cells separated by a filter paper membrane during the co-cultivation phase [5]. It was proposed that the feeder layer enhances the *Agrobacterium* transformation efficiency. But, like Van Eck et al. [20], we found that we could dispense with the feeder layer altogether. Some publications have attempted to perform multi-variate analysis as there are many, many parameters to cover [40]; however, you may find that scanning the literature and testing major parameters before moving to minor parameters may be less time-consuming. Do not be put off. It is clear in the literature and from our own work that a continued interest in streamlining, simplifying, and being aware of other variables has enabled us to continually provide a reliable transformation service to our research institute and collaborators.

Differences in bacterial infection methods between our protocols are minimal. The method of choice for *Agrobacterium* infection is overwhelmingly co-cultivation for all our protocols and the majority in the literature. Everyone needs to experiment with their co-cultivation conditions and the *Agrobacterium* growth conditions. The majority of papers tend to target a 1 to 2 day overnight stationary phase, with an OD_600_ 0.6–1.0 yielding good results (here and references herein). We employ two-day incubations for all of our protocols, with minimal exposure to the Agrobacteria co-cultivation protocols as they evolved. As mentioned in the introduction, Agrobacteria infect wounded tissue, which they need for entry, and take advantage of the cellular changes that occur in wounded tissue for cellular repair and cells in an active cell cycle phase [41]. Phenolic compounds are released by wounded cell wall material, and acetosyringone is one such compound that is sensed by an *Agrobacterium* two-component signaling system to ramp up its virulence for plant infection. Therefore, it is no surprise that the addition of acetosyringone is helpful and the various effective concentrations (100–500 µM) are likely due to media differences, plant genotypes, and culture conditions (the various references herein). It is interesting that some protocols experimented with very short, “direct wound infection” by just cutting tissue with infected scalpels [6] or stabbing with infected needles [42], both yielding infection rates comparable to co-cultivation. We have not tried this out ourselves. If co-cultivation overgrowth is still an issue, you may consider dropping the temperature to reduce the rate of bacterial growth [43].

Although we only use very few cultivars, our protocols can likely be directly used with others or with only minor modifications, as many of the progenitor protocols explored many different cultivars. For example, tomato cv. Money maker, Yellow Pear, Rio Grande, Micro-Tom, Great White, plus more [20]. *N. tabacum* cultivars Xanthi, Samsum [6]. Potato cultivars Bintje, Berotina Russet Burbank, and Désirée were all transformable with nearly the same protocol [32].

Plant regeneration from protoplasts is also possible. One concern is the idea of genomic instability, like aneuploidy or structural chromosomal changes, that could potentially arise from tissue culture techniques. In a study by Fossi et al., they compared plant regeneration from protoplasts to tissue culture [23]. In it, they observed that 100% of the protoplast-regenerated plants had DNA aberrations, while only 18% of the tissue culture plants had large-scale copy number changes. Thus, 82% of all the plants did not, indicating that tissue culture is a viable method for the production of transgenic plants and crops.

Many protocols that have been developed for one species can be applied to another. For example, tomato transformation [19,20] was adapted to another Solanaceae species, *Datura stramonium*, with minimal changes [44]. One challenge is sometimes rooting. The presence of cytokinin prevents rooting [45] and is best left out of all rooting media [46]. Occasionally, for other species, a little bit of an auxin helps (our protocols, [44]), but it should be tested only when your particular species does not produce any roots at all. For some *Nicotiana* species like *N. edwardsonii*, different hormone concentrations were needed [46]. If you have a lot of browning, then more than likely this is due to an overproduction of ethylene, which eventually kills the plant tissue when working in tissue culture [32,43], and its control with AgNO_3_ was shown to be necessary for *Capsicum* ssp. [47], peanut, and several others (see references in [43]). One major observation is that you should add AgNO_3_ after autoclaving, as autoclaving leads to toxic media that kill the plants [32]. On the other hand, inhibiting ethylene production altogether is not a good idea either as root growth/production can be inhibited [32], which might be related to its role in root development and signaling [48].

When considering other species, do not forget that the Agrobacteria type might play a role. *Nicotiana glauca* was best infected with octopine-type Agrobacteria, like LBA4404 [49]. In the literature mentioned throughout, the octopine strain LBA4404 is very popular and used to great success with all of the species given here and many others. However, it has been noted that you should periodically check your plasmid DNA regularly for recombination events, as LBA4404 is recA-positive [20], but this is only a minor nuisance compared to its utility.

Over the years, many different types of plants have been successfully transformed, and many of these protocols are available. These include rapeseeds; peanuts; soybeans; lettuce; various monocots like barley, maize, and rice; other dicots; and many, many more [2,3,9,10]. And although the most common antibiotic markers are kanamycin and hygromycin, or herbicide, Phosphinothricin (BASTA)/bialaphos, others like blasticidin S, mannose as sole carbon source, Bleomycin, phleomycin, sulfonamide, methotrexate, chlorsulfuron, cyanamide, D-xylose as sole carbon source, plus many more exist [50]. Many of these have still only been tested in a few species to this day, and as such, it is worth remembering that there are more options for selection markers. Of course, with CRISPR/Cas9, genome editing can be marker-free if properly screened, and some reports for site-directed mutagenesis integration/modification are appearing [51].

## 5. Reagent Setup

In general, when using plant media, do not use media that is older than one month due to a decrease in the activity of hormones and antibiotics. If a fungal infection occurs, you may consider adding amphotericin (5 mg/L) to the medium, but generally, it is better not to open the contaminated jars in order to avoid the diffusion of fungal spores; the best step is to just autoclave the contaminated jars. Note: never open contaminated jars in the cultivation chambers, as there is a risk that the air supply and filters could become contaminated and would be extremely difficult to clean afterwards. Major stock solutions with chelated iron should be kept at 4 °C. Hormones are best stored a −20 °C or maximally a month at 4 °C. Media plates can be stored up to one month at 4 °C, after which they should be discarded.

### 5.1. Agrobacteria Media

#### 5.1.1. YEB 1x

Add 5 g/L beef extract, 1 g/L yeast extract, 5 g/L peptone, 5 g/L sucrose, and 0.49 g/L MgSO4•7H_2_O. For plates, add 15 g/L Bacto Agar (Difco) directly into the bottle. Autoclave (121 °C, 20 min).

#### 5.1.2. (Low-Salt) lsLB/LB 1x

Add 10 g/L tryptone, 5 g/L yeast extract, and 10 g/L NaCl (LB) or 5 g/L NaCl for low-salt medium (lsLB). For plates, add 15 g/L Bacto Agar (Difco) directly into the bottle. Autoclave (121 °C, 20 min).

#### 5.1.3. Bacteria-Growth Medium (BGM) 1x

Add 10 g/L yeast-extract, 10 g/L peptone, and 5 g/L NaCl. Autoclave (121 °C, 20 min).

#### 5.1.4. Acetosyringone 400 mM

Dissolve in DMSO. Store in 1 mL aliquots at −20 °C in sterile Eppendorf tubes.

#### 5.1.5. Antibiotics for Bacteria

Ampicillin 100 mg/L (stock 100 mg/mL ddH_2_O, filter-sterilize at 0.2 µm).

Rifamycin 100 mg/L (stock 50 mg/mL DMSO).

Kanamycin 25 mg/L (Stock 50 mg/mL ddH_2_O, filter-sterilize at 0.2 µm).

Gentamycin 40 mg/L (stock 10 mg/mL ddH_2_O, filter-sterilize at 0.2 µm).

Note: Add antibiotics to a 60 °C warm medium, stir well, and pour plates immediately. If plates contain antibiotics, they should not be kept longer than a month.

### 5.2. Hormones and Antibiotics for Plants

#### 5.2.1. Amphotericin B Fungicide (ABF) 5 mg/mL

Dissolve in DMSO. Filter-sterilize at 0.2 µm and store in 1 mL aliquots at −20 °C in sterile Eppendorf tubes. Note: store powder at 4 °C, durable for 3 days at 37 °C.

#### 5.2.2. Cefotaxime Sodium (Cef) 250 mg/mL

Dissolve in ddH_2_O. Filter-sterilize at 0.2 µm and store in 1 mL aliquots at −20 °C in sterile Eppendorf tubes.

#### 5.2.3. Basta Herbicide (PPT) 20 g/L

Dilute in ddH_2_O. Filter-sterilize at 0.2 µm and store at RT.

#### 5.2.4. 6-Benzylaminopurine (BAP) 1 mg/mL

Dissolve 40 mg in 1 mL 1 N NaOH and add sterile ddH_2_O up to 40 mL. Filter-sterilize at 0.2 µm and store in 1 mL aliquots at −20 °C in sterile Eppendorf tubes.

#### 5.2.5. Indole-3-Acetic Acid (IAA) 1 mg/mL

Dissolve in 1/10 final vol. in 100% ethanol, and then 9/10 final vol. sterile ddH_2_O. Filter-sterilize at 0.2 µm and store in 1 mL aliquots at −20 °C in sterile Eppendorf tubes.

#### 5.2.6. Gibberellic Acid (GA_3_) 20 µg/mL

Dissolve in ddH_2_O; filter-sterilize at 0.2 µm; and store in 1 mL aliquots at −20 °C in sterile Eppendorf tubes.

#### 5.2.7. Hygromycin B (Hyg) 12 mg/L or 15 mg/L

Dilute in sterile ddH_2_O. Filter-sterilize at 0.2 µm and store in 1 mL aliquots at −4 °C in sterile Eppendorf tubes; freezing should be avoided.

#### 5.2.8. Naphtalenacetic Acid (NAA) 1 mg/mL

Dissolve in 1/10 final vol. 1 N NaOH and then add 9/10 final vol. ddH_2_O. Filter-sterilize at 0.2 µm and store in 1 mL aliquots at −20 °C in sterile Eppendorf tubes.

#### 5.2.9. Kanamycin Monosulfate (Kan) 50 mg/mL

Dilute in ddH_2_O (note: 60 mg kanamycin salt contains approx. 50 mg kanamycin). Filter-sterilize at 0.2 µm and store in 1 mL aliquots at −20 °C in sterile Eppendorf tubes.

#### 5.2.10. Ticarcillin Disodium and Clavulanate Potassium (15:1) (TiCla) 250 mg/mL

Dilute in ddH_2_O. Filter-sterilize at 0.2 µm and store in 1 mL aliquots at −20 °C in sterile Eppendorf tubes.

#### 5.2.11. trans-Zeatin (t-Z) 1 mg/mL

Dissolve in 1/10 vol. 1 *N* NaOH, then 9/10 vol. ddH_2_O. Filter-sterilize at 0.2 µm and store in 1 mL aliquots at −20 °C in sterile Eppendorf tubes.

#### 5.2.12. Vancomycin Hydrochloride (Van) 125 mg/mL

Dilute 5 g in 40 mL sterile ddH_2_O. Filter-sterilize at 0.2 µm and store in 4 mL aliquots at −20 °C in sterile Falcon tubes.

#### 5.2.13. Zeatin Riboside (ZR) 1.4 mg/mL

Dissolve in 1/10 vol. 0.1 N HCl, and then add 9/10 vol. ddH_2_O. Filter-sterilize at 0.2 µm and store in 1 mL aliquots at −20 °C in sterile Eppendorf tubes.

### 5.3. Plant Media for Tomato

#### 5.3.1. NPT Vitamins Mix (NPT)

Mix all in ddH_2_O: 10 mg/mL Thiamine-HCl; 1 mg/mL Nicotine acid; and 1 mg/mL Pyridoxine-HCl. Filter-sterilize at 0.2 µm and store in 1 mL aliquots at −20 °C in sterile Eppendorf tubes.

#### 5.3.2. Tomato Medium (ToMe) 1 L

Dissolve in 900 mL ddH_2_O: 4.3 g Murashige & Skoog Salts, 30 g sucrose, 100 mg myo-Inositol, and 1 mL NPT Vitamins. Adjust pH to 5.8 with 1 M KOH (about 6 droplets); bring to 1 L; and add 7 g Daishin agar to the bottle. Autoclave (117 °C, 20 min). Stir well and pour immediately into larger jars under a clean bench until solidified. Can be stored at 4 °C until needed.

#### 5.3.3. Tomato Liquid Germination Medium (ToLGM) 500 mL

Dissolve in 450 mL ddH_2_O: 2.15 g Murashige & Skoog Salts, 15 g sucrose, 50 mg myo-Inositol, and 0.5 mL NPT Vitamins. Adjust pH to 5.8 with 1 M KOH (1–2 droplets) and bring to 0.5 L. Autoclave (117 °C, 20 min).

#### 5.3.4. Tomato Conditioning Medium (ToCM) 1 L

ToMe Medium (Section 5.3.2) cooled to 60 °C (hand warm); add to final concentration 0.1 mg/L BAP and 1 mg/L NAA; stir well and pour immediately into Petri dishes (9 cm Ø); and leave under a clean bench until solidified.

#### 5.3.5. Tomato Selection Medium (toSM) 1 L

ToMe Medium (Section 5.3.2) cooled to 60 °C (hand warm); add to final concentration 1 mg/L t-Z; against *Agrobacterium*, 250 mg/L TiCla; and T-DNA selection antibiotic (e.g., either 35–100 * mg/L Kan, 2 mg/L PPT or 6 mg/L Hyg). Stir well and pour immediately into smaller jars under a clean bench until solidified. *Kanamycin seems to work better in tomato if the concentration is increased over time.

#### 5.3.6. Tomato Rooting Medium (ToRM) 1 L

ToMe Medium (Section 5.3.2) cooled to 60 °C (hand warm); add to final concentration 0.1 mg/L IAA; against *Agrobacterium* 500 mg/L Van; and T-DNA selection antibiotic (e.g., either Kan 20 * mg/L, 2 mg/L PPT, or 6 mg/L Hyg). Stir well and pour immediately into jars under a clean bench until solidified. *Kan seems to work better if the concentration is now reduced; if there are any problems with rooting, leave out Kan or PPT.

### 5.4. Plant Media for Potato

#### 5.4.1. GmNPT Vitamins Mix (GmNPT) 200x

Mix together in ddH_2_O: 20 mg/L Thiamine-HCl; 100 mg/L Nicotine acid; 100 mg/L Pyridoxine-HCl; 400 mg/L Glycine; and 20 g/L myo-Inositol. Filter-sterilize at 0.2 µm and store in 25 mL aliquots at −20 °C.

#### 5.4.2. Potato MS Medium (PoMS/PoSMS) 1 L

Dissolve in 900 mL ddH_2_O: 4.31 g MS-salts, 5 mL GmNPT vitamin mix. For PoSMS, add 20 g sucrose. Adjust pH to 5.7–5.8, with 1 M KOH (8–10 droplets). For solid PoMS/PoSMS, add 7 g Daishin agar to 1 L. Fill up to 1 L and Autoclave (117 °C, 20 min).

#### 5.4.3. Potato Growth Medium (PoGM) 1 L

Dissolve in 900 mL ddH_2_O: 4.31 g MS-salts; 16 g glucose; and 5 mL GmNPT vitamin mix. Adjust pH to 5.7–5.8 with 1 M KOH (8–10 droplets). Add 7 g Daishin agar to the bottle. Autoclave (117 °C, 20 min). Stir well and pour immediately into larger jars under a clean bench until solidified. Can be stored at 4 °C until needed.

#### 5.4.4. Potato Callus Induction Medium (PoCIM) 1 L

PoMG Medium (Section 5.4.3) cooled to 60 °C (hand warm); add to final concentration 5 mg/L NAA, 0.1 mg/L BAP, 250 mg/L TiCla, and T-DNA selection antibiotic (e.g., either 50 mg/L Kan, 2 mg/L PPT, or 1 mg/L Hyg).

#### 5.4.5. Potato Selection Medium (PoSM) 1 L

PoMG Medium (Section 5.4.3) cooled to 60 °C (hand warm); add to final concentration 1.4 mg/L Zeatin riboside, 20 µg/L GA_3_, 20 mg/L NAA, 250 mg/L TiCla, and T-DNA selection antibiotic (e.g., either 50 mg/L Kan, 2 mg/L PPT, or 3 * mg/L Hyg). * Is increased.

#### 5.4.6. Potato Rooting Medium (PoRM) 1 L

PoMG Medium (Section 5.4.3) cooled to 60 °C (hand warm); add to final concentration 250 mg/L TiCla and T-DNA selection antibiotic (e.g., either 50 mg/L Kan, 2 mg/L PPT, or 3 mg/L Hyg). Note: Absence of hormones allows rooting to occur.

### 5.5. Plant Media for Nicotiana

#### 5.5.1. 0.1%. Agarose

Dissolve 100 mg of Bacto Agar agarose in 100 mL of pure water and autoclave. It should have a gel-like consistency.

#### 5.5.2. Nicotiana Growth Medium (NicoGM) 1 L

Dissolve in 900 mL ddH_2_O: add 4.31 g MS-salt; 20 g sucrose; 5 mL GmNPT (Section 5.4.1) vitamin mix; adjust pH to 5.7–5.8 with 1 M KOH (about 8–10 droplets); fill up to 1 L. Add 7 g Daishin agar to the bottle. Autoclave (117 °C, 20 min). Stir well and pour immediately into larger jars under a clean bench until solidified. Can be stored at 4 °C until needed.

#### 5.5.3. Nicotiana Selection Medium (NicoSM) 1L

Dissolve in 900 mL ddH_2_O: add 4.31 g MS-salt; 16 g glucose; 5 mL GmNPT (Section 5.4.1) vitamin mix; adjust pH to 5.7–5.8 with 1 M KOH (about 8–10 droplets); fill up to 1 L. Add 7 g Daishin agar to the bottle. Autoclave (117 °C, 20 min). Can be stored at 4 °C until needed. Once cooled to 60 °C (hand warm), add to the final concentration 1 mg/L BAP, 0.2 mg/L NAA, *Agrobacterium* antibiotic 500 mg/L Cef, and T-DNA selection antibiotic (e.g., either 50 mg/L Kan, 4 mg/L PPT, or 15 mg/L Hyg). Stir well and pour immediately into smaller jars under a clean bench until solidified.

#### 5.5.4. Nicotiana Rooting Medium (NicoRM) 1 L

NicoGM solid medium (Section 5.5.2) cooled to 60 °C (hand warm); add to final concentration *Agrobacterium* antibiotic 500 mg/L Cef, and T-DNA selection antibiotic (e.g., either 50 mg/L Kan, 4 mg/L PPT or 15 mg/L Hyg). Stir well and pour immediately into larger jars under a clean bench until solidified. Note: Absence of hormones allows rooting to occur.

## Data Availability

Data is available upon request.
